# Impact of COVID-19 on Eye Care in Spain during the First Phase of the Pandemic

**DOI:** 10.3390/jcm10184087

**Published:** 2021-09-10

**Authors:** Carmen Antía Rodríguez-Fernández, María Varela-Agra, Lucía Pérez-Roldán, Ana Álvarez-Reguera, Cristina Martínez-Reglero, Ana Campo-Gesto

**Affiliations:** 1 Ophthalmology Service, Complejo Hospitalario Universitario de Vigo, 36313 Vigo, Spain; Lucia.Perez.Roldan@sergas.es (L.P.-R.); Ana.Alvarez.Reguera@sergas.es (A.Á.-R.); Ana.Campo.Gesto@sergas.es (A.C.-G.); 2 Methodology and Statistics Unit, Galicia Sur Health Research Institute (IIS Galicia Sur), SERGAS-UVIGO, 36313 Vigo, Spain; cristina.martinez@iisgaliciasur.es

**Keywords:** coronavirus, COVID-19, mental health, ophthalmology, residency program, Spain

## Abstract

Background: The declaration of the first state of alarm for COVID-19 in March 2020 provoked changes in ophthalmological care. The objective of this study was to assess its impact on reorganising care activities, the mental health of ophthalmologists and the training of residents. Methods: We sent an anonymous online questionnaire between August and October 2020 to consultant ophthalmologists and residents who were active during the state of alarm in Spain. We used Google Forms^®^ software for data collection. We analysed responses according to the degree of regional impact. Results: We received a total of 328 responses from the 17 Autonomous Communities. We saw that 99.4% of respondents changed their work activities with 50% reductions in surgery (94.5%) and consultations (93.0%). Furthermore, 58.8% of respondents reported increased anxiety, and 29.9% transferred to support other services, with this number reaching 49.6% in the hardest-hit regions. Training programs were greatly reduced in external consultations (90.7%), and surgical training was completely cancelled (100%). Additionally, 56.5% of trainees wanted to prolong their residence periods. Conclusions: The first wave of the pandemic produced significant changes in ophthalmology services, and these changes were more pronounced in the most affected regions. It caused a negative psychological impact on a high rate of respondents and an interruption of the training of ophthalmology residents. Predictably, the negative consequences of this delay in ophthalmological care on patients will be uneven between regions.

## 1. Introduction

The state of alarm was established in Spain between 14th March and 21st June 2020 in the wake of the SARS-CoV-2 virus health emergency. This virus causes the disease referred to as COVID-19 (an acronym for Corona VIrus Disease-2019) [1], which was declared a pandemic by the World Health Organization (WHO) in March 2020 [2].

During this period, there was a notable change in the functioning of ophthalmology services. These changes followed the recommendations set up by the Spanish Society of Ophthalmology, ref. [3], and other national and international societies. Ophthalmological examinations were considered risky because of the high transmission capacity of SARS-CoV2 and the closeness between the physician and the patient, despite protective measures [3]. Therefore, any non-urgent activity was delayed, favouring non-in-person consultation to remotely evaluate the risk/benefit balance according to groups of pathologies [4]. Thus, many patients were rescheduled to a later date that was deemed safe and with less hospital overload [5]. Only urgent consultations and check-ups, as well as urgent surgical interventions, were maintained [6]. An estimated 45,684 people lost their lives due to COVID-19 between March and May 2020 in Spain [7]. The impact was highly unequal among autonomous communities, with the highest death rates due to identified virus per 100,000 inhabitants being seen in Castilla-La Mancha (160.8) and Madrid (150.6), and the lowest in the Canary Islands (7.5) and Murcia (9.3) [8].

Past evidence exists of the negative effect of pandemics on mental health [9]. Following the expansion of SARS-CoV2, the impact of the pandemic on health professionals [10,11] as well as on resident ophthalmology training programmes, ref. [12], among other surgical specialties, ref. [13], were analysed.

Our survey was designed to assess the impact of the global public health crisis due to the new coronavirus pandemic on ophthalmological care and ophthalmology professionals in Spain. We assessed differences according to the degree of geographical impact.

## 2. Material and Methods

We distributed the link to an online questionnaire via email and social networks between August and October 2020, conducted in collaboration with Ophthalmological Societies. The link was closed before the declaration of the second state of alarm (25 October). We surveyed consultant ophthalmologists and trainees who had been working in Spain between 14 March and 11 May 2020, which was the day on which the de-escalation began.

This cross-sectional observational analytic study was conducted according to the guidelines of the Declaration of Helsinki. Research ethics authority, namely Galician Clinical Research Ethics Committee, confirmed this type of study is automatically exempt from requiring ethics approval. No personal data were collected, and the data are reported in grouped format, such that the individual identity of the respondents was kept anonymous and secure. The survey was answered anonymously, ophthalmologists received no incentives and were not forced to participate. Prior to the completion of the questionnaire, informed consent was obtained from all participants. We used Google Forms^®^ software for data collection.

We divided the 44 questions in the survey into five sections. The first section asked about organisational changes in the service during the state of alarm. The second focused on mental health. The third collected demographic data, autonomous community, professional category, hospital management and service size. The fourth section, which was aimed at residents, assessed the impact on their training. In the last section, and with a view to the future, we asked about the perception of safety following the changes made.

After analysing the data available during the first wave at the Carlos III Institute of Health, [8] we classified the autonomous communities into two groups with reference to the national global average of excess mortality according to the degree of impact by COVID-19. These “impact” groups were: group A (the least affected communities: Andalusia, Asturias, the Balearic Islands, the Canary Islands, Cantabria, Extremadura, Galicia, Murcia, The Basque Country and Valencia,) and Group B (the most affected communities: Aragon, Castilla-La Mancha, Castile and León, Catalonia, La Rioja, Madrid and Navarre).

We performed a descriptive analysis of the data and expressed qualitative variables as frequencies and percentages, and quantitative variables as mean and standard deviation (SD). We used the chi-square, T-student or Mann–Whitney tests to study the differences in questionnaire responses based on the “impact” group to which the autonomous community belonged. We considered *p* < 0.05 to be statistically significant and analysed the data using SPSS version 19.

## 3. Results

### 3.1. Demographics

A total of 328 ophthalmologists completed the survey, 220 consultants and 108 trainees. Of these, 192 were women and 134 men, and the average age was 40.6 ± 12.8 years old. Table 1 gives the demographic characteristics of respondents and hospitals. It is noteworthy that most respondents worked in public hospitals (89.6%). We received replies from all autonomous communities.

### 3.2. Impact on Clinical Activity and Organisational Changes in the Service

Almost all ophthalmologists reported changes in their work activity (99.4%). They reported more than 50% reductions in surgery (94.5%) and consultations (93.0%), with no differences being seen between regions (Table 2).

Most (93.6%) considered that the number of emergencies decreased by more than 50% and that only urgent surgeries (63.4%) were performed, with greater restrictions in the most affected communities.

Transferring ophthalmologists to other services was greater in the most affected communities (16.3% vs. 49.6%, *p* < 0.001), with no differences between consultants and residents (29.1% vs. 31.5% respectively, *p* = 0.657) being seen. Nearly half of the ophthalmologists transferred (49.5%) provided physical support to services with COVID-19 patients, 48.5% made phone calls for diagnostic test results or contact tracing and the remaining 2% handled organisational issues.

### 3.3. Impact on Mental Health

Most ophthalmologists referred to feeling unprotected (91.2%), increasing anxiety levels (58.8%), worsening sleep quality (53.7%) and starting anxiolytic or sleep-inducing treatment (12.5%). There were no differences between groups (Table 3). Overall, we did not see any differences by gender or professional category.

### 3.4. Impact on Residents in Training

A total of 108 residents answered the survey (Table 4). Their healthcare activities, both in the consultation and operating room, were largely cancelled or postponed in 90.7% and 100% of cases, respectively. The vast majority (95.4%) considered that changes made during the state of alarm would adversely affect their training (rated two or more on a scale of 0 to 4), and 56.5% wanted to extend their residency periods. We did not see differences between groups.

### 3.5. Perception of Safety

Professionals were asked about the changes being implemented at the time of the survey. We found that 77.7% did not consider themselves to be prepared to face a second wave, and 88.1% said that the measures were insufficient. We did not see any differences between communities (*p*-Value 0.383 and 0.115, respectively).

## 4. Discussion

COVID-19 has led to a change in hospital organisation, which has affected eye care.

### 4.1. Care Modifications

The way of working for most of the ophthalmologists surveyed (99.4%) was modified, with a decrease in care and surgical activity motivated by the recommendations of scientific societies at the beginning of the pandemic [3]. This reduction had a particularly high impact on waiting lists during the first half of 2020, ref. [14], compared to the same period in 2019, with an approximately 100% increase in average waiting times, in all regions. Similarly, a 79% reduction in eye consultations during the first wave was reported in the United States. This was the greatest reduction for any medical or surgical specialty [15].

Non-in-person consultations were performed by 85.1% of our respondents. Arntz and collaborators [16] demonstrated that, despite its limitations, telemedicine in ophthalmology (TMO) can be a useful tool in exceptional situations, such as the pandemic, for eye pathology screening, reduction of in-person consultations and patient stress relief.

Fear of going to the hospital lead to reduced use of non-respiratory pathology emergency services during the first weeks of the pandemic. This coincided with periods of higher COVID-19 mortality rates [17]. In addition, 93.6% of respondents reported a great decrease in the number of patients requiring urgent eye care. García Lorente and collaborators [18] recorded an 82% decrease during the period that the state of alarm lasted compared to the same period in previous years. They report fewer consultations for not only non-urgent pathologies such as dry eyes, but also for pathologies that should not be delayed such as retinal detachments.

The reduction in ophthalmological activity was greater in the most affected areas in all respects (although this was not always statistically significant, Table 2). Delayed medical care during the pandemic could lead to aggravating other pathologies that are independent of COVID-19 in the coming months, with this impact probably being greater in the regions with more restrictions, as our respondents anticipate. This may include ophthalmological pathologies, especially macular diseases in need of intravitreal therapy, the delay of which could lead to irreversible consequences [19]. Authors working in a tertiary centre in Italy evidenced a reduction of up to 91.7% in intravitreal injections during the quarantine [20]. Switching from monthly anti-vascular endothelial growth factor (VEGF) injections to intravitreal dexamethasone implant in eligible patients has been suggested in order to reduce the burden of injections for hospitals [20,21].

In line with population data, there was a statistically significant higher incidence of COVID-19 among ophthalmologists in the more affected regions. Despite the importance of asymptomatic screening, especially of health workers [22], recommendations for isolation and contact tracing were less prevalent in these regions. Protocols may not have been properly implemented because of the increased need for staff, as reflected in our results, with more ophthalmologist collaborations in other services in these communities.

### 4.2. Mental Health

Mental health conditions have previously been described in the general population as a result of the pandemic. Kantor and collaborators, ref. [23], with a thousand U.S. participants, find anxiety (52.1%) and depressive symptoms (47.3%). Higher levels of anxiety and hopelessness have been found in health professionals compared to the general population, refs. [24,25], with the greatest impact in young women [10,23].

The psychological impact on ophthalmologists in Spain has been very high. Most respondents expressed an increase in anxiety and worsening sleep quality. Wang and collaborators [26] observe a combined prevalence of 38% anxiety, depression and insomnia in Hubei’s health workers. Previous studies also showed the negative impact of COVID-19 on the mental health of ophthalmologists in India [27], with high depression levels (32.6%).

Only 5.5% reported total availability of Personal Protective Equipment (PPEs), with greater deficiencies being seen in the least affected regions. The feeling of unprotection stood at 91.2%, and most respondents (77.7%) considered that they were unprepared for future waves. Similar rates were obtained in a survey in the Cairo metropolitan area (Egypt) [28], where 82% of the ophthalmologists were not feeling safe during their practice. Precarious working conditions during the pandemic have been associated with a negative impact on the mental health of health workers in other areas such as primary care [11].

### 4.3. Resident Training

Ophthalmology residents saw that training in their specialty was highly reduced, and some even saw it eliminated. Many respondents (95.4%) felt that the pandemic would adversely affect their training.

A recently published report [29] coincides with these results, with 80.5% of our country’s trainees considering that their training was impaired during the pandemic, 97.3% lost surgical teaching and 59.9% would opt for residency expansion. Pertile and collaborators [13] study the impact on surgical resident training programs in Italy. They report that most of the trainees experienced a reduction or complete interruption of their training.

On analysing ophthalmology residents from multiple countries, Ferrara’s team [12] found that 55.2% of residents reported severe impact on their training, with a decrease of more than 50% in the clinical activity being generally reported (76.4%) and more than a 75% decrease in surgical practices (74.6%). In the United Kingdom (UK) [30], 86% of the trainees were concerned about the impact of the COVID-19 pandemic, with cataract surgery as the biggest concern. In India [31], the impact on surgical training of ophthalmologists has also been negative (80.7%), with increasing stress levels (54.8%) being reported. Another Indian survey shows 52.8% of ophthalmologists feeling the negative impact of COVID-19 on their training or profession [27].

More than a third of trainees moved to other areas, and 15.7% had duties in another service, with the highest number of displaced residents being seen in the most affected regions. This high rate would explain why more than half of the residents wanted to extend their residency periods. In Italy, with the transferral of 12.5% of surgical residents [13], 3.2% voluntarily decided to discontinue their training. In the UK, 39% of ophthalmology trainees were redeployed to specialities outside ophthalmology [30].

At the same time, in response to the cessation of ophthalmological activity and probably due to stay-at-home orders, the number of hours devoted to home study increased from previous stages in 82.5% of respondents, similar to Egyptian trainees (80%) [28]. Less time was spent studying in the most affected communities. This was in line with the greater need to help other specialties and probably with less time being available to study. As was reported in other countries, Ferrara and collaborators [12] highlight high participation in online case discussions (91.7%), surgical videos (85.7%) and online classes (67.7%). In India, ref. [31], 75.7% of residents found online classes and webinars useful. Furthermore, UK trainees had positive opinions on webinars, with a desire for continuation once the pandemic is over [30]. Collaboration between different centres and working groups even made it possible to unify ophthalmology training programs online in some large areas such as New York [32].

### 4.4. Strengths and Limitations

We present the first study developed in Spain that specifically investigated the impact of COVID-19 on the activity of ophthalmology professionals, and which included both consultants and residents in training. We performed a comparative analysis between different geographical areas in Spain.

The limitations of this study include its retrospective character and small sample size.

Further studies would be needed to assess whether teleconsultation or the delay of ophthalmological treatments, among other measures, will have an impact, and to what extent this impact may be, on the patients concerned. A new survey after the second wave would allow us to assess whether the problems identified during the first wave have been resolved, such as the availability of PPEs, the deterioration in the training of residents and the need for collaboration of ophthalmologists in other services.

## 5. Conclusions

COVID-19 has led to a shift from the classic model of in-person ophthalmological care to predominantly telematic care. This was done to slow down the transmission of the virus.

SARS-CoV-2 has conditioned the complete reorganisation of the hospital, and not just for the specialties that directly treat COVID-19. Modifications during the first wave were greater in the hardest-hit communities. This was demonstrated by uneven hospital overloads. Respondents considered these modifications to be insufficient to deal with future waves.

The situation forced the transfer of ophthalmologists and provoked a delay of scheduled consultations and elective surgeries. This, in turn, hindered the management of ophthalmological patients and demonstrated that this strategy could not be maintained over time. In the long term, the consequences will not only be for the patients concerned but will also have a negative psychological and professional impact on the ophthalmology professionals. The training of residents has been particularly impaired, which could lead to a shortfall in the quality of care in the future.

## Figures and Tables

**Table 1:** Demographic Characteristics.

		Total No. 328	Percentage (%)
Sex	Man	134	40.9%
	Woman	192	58.5%
	Unanswered	2	0.6%
Age	Mean ± SD	40.6 ± 12.8	
Professional category	Consultant	220	67.1%
	Resident	108	32.9%
Hospital management	Public	294	89.6%
	Private	34	10.4%
Service size	<10 consultants	51	15.5%
	10–20 consultants	126	38.4%
	>20 consultants	151	46.1%

**Table 2:** COVID-19 impact on care activity.

Group (*n*, Percentage (%))		Total No. 328	Group A No. 196	Group B No. 129	*p*-Value
Changed work activity	Yes	326 (99.4%)	194 (99.0%)	129 (100.0%)	*p* = 0.250
	No	2 (0.6%)	2 (1.0%)	0 (0.0%)	
Reduced in-person consultation	Yes >50%	305 (93.0%)	180 (91.8%)	124 (96.1%)	*p* = 0.151
	Yes 25–50%	17 (5.2%)	14 (7.1%)	3 (2.3%)	
	No (<25%)	6 (1.8%)	2 (1.0%)	2 (1.6%)	
Non-in-person consultation in non-urgent patients	Yes	279 (85.1%)	167 (85.2%)	111 (86.0%)	*p* = 0.833
	No	49 (14.9%)	29 (14.8%)	18 (14.0%)	
Reduced surgical activity	Yes >50%	310 (94.5%)	182 (92.9%)	125 (96.9%)	*p* = 0.078
	Yes, 25–50%	17 (5.2%)	14 (7.1%)	3 (2.3%)	
	No (<25%)	1 (0.3%)	0 (0.0%)	1 (0.8%)	
Type of surgery performed	Only urgent	208 (63.4%)	103 (52.6%)	102 (79.1%)	***p* < 0.001**
	Urgent and preferred	118 (36.0%)	91 (46.4%)	27 (20.9%)	
	Elective	2 (0.6%)	2 (1.0%)	0 (0.0%)	
Performed pre-surgical PCR tests	Yes, everyone	274 (83.5%)	151 (77.0%)	120 (93.0%)	***p* < 0.001**
	Only admitted patients or with anaesthesia	28 (8.5%)	25 (12.8%)	3 (2.3%)	
	No/only suspected	26 (7.9%)	20 (10.2%)	6 (4.7%)	
Decreased numbers of emergencies	Yes > 50%	307 (93.6%)	178 (90.8%)	126 (97.7%)	***p* = 0.043**
	Yes, mild < 50%	17 (5.2%)	15 (7.7%)	2 (1.6%)	
	No	4 (1.2%)	3 (1.5%)	1 (0.8%)	
I work in another service	Yes	98 (29.9%)	32 (16.3%)	64 (49.6%)	***p* < 0.001**
	No	230 (70.1%)	164 (83.7%)	65 (50.4%)	
Own diagnosis of COVID-19	Yes	24 (7.3%)	7 (3.6%)	17 (13.2%)	***p* = 0.001**
	No	304 (92.7%)	189 (96.4%)	112 (86.8%)	
Diagnosis of COVID-19 from a partner and contact tracing	Yes, with contact tracing	147 (16.8%)	31 (15.8%)	23 (17.8%)	***p* < 0.001**
	Yes, without isolation or contact tracing	55 (44.8%)	61 (31.1%)	84 (65.1%)	
	No	126 (38.4%)	104 (53.1%)	22 (17.1%)	
Availability of Personal Protective Equipment (PPEs) in ophthalmological consultation	Non-existent or highly insufficient	49 (14.9%)	39 (19.9%)	8 (6.2%)	***p* = 0.001**
	Only + patients	33 (10.1%)	24 (12.2%)	9 (7.0%)	
	One-off or initial deficiencies	228 (69.5%)	122 (62.2%)	105 (81.4%)	
	Yes, total without deficiencies	18 (5.5%)	11 (5.6%)	7 (5.4%)	

**Table 3:** Impact of COVID-19 on mental health.

Group (*n*, Percentage (%))		Total No. 328	Group A No. 196	Group B No. 129	*p*-Value
Feeling unprotected	Yes, almost constantly	124 (37.8%)	83 (42.3%)	39 (30.2%)	*p* = 0.085
	Yes, at the beginning or at specific times	175 (53.4%)	98 (50.0%)	77 (59.7%)	
	No	29 (8.8%)	15 (7.7%)	13 (10.1%)	
Altered level of anxiety	Yes, higher than before	193 (58.8%)	117 (59.7%)	76 (58.9%)	*p* = 0.220
	No	114 (34.8%)	70 (35.7%)	41 (31.8%)	
	Yes, lower than before	21 (6.4%)	9 (4.6%)	12 (9.3%)	
Altered sleep quality	Yes, negatively	176 (53.7%)	103 (52.6%)	73 (56.6%)	*p* = 0.064
	No	130 (39.6%)	84 (42.9%)	43 (33.3%)	
	Yes, positively	22 (6.7%)	9 (4.6%)	13 (10.1%)	
Initiation of anxiolytic or sleep-inducing treatment	Yes	41 (12.5%)	22 (11.2%)	19 (14.7%)	*p* = 0.352
	No	287 (87.5%)	174 (88.8%)	110 (85.3%)	
Subjective feeling of worse care and negative effects on the ophthalmological patients	Yes, in many patients	185 (56.4%)	96 (49.0%)	86 (66.7%)	***p* = 0.002**
	Yes, only in serious patients	115 (35.1%)	77 (39.3%)	38 (29.5%)	
	No	28 (8.5%)	23 (11.7%)	5 (3.9%)	

**Table 4:** Impact of COVID-19 on resident training.

Group (*n*, Percentage (%))		Total No. 108	Group A No. 53	Group B No. 54	*p*-Value
Loss of eye care activity	Every day or almost every day	98 (90.7%)	49 (92.5%)	48 (88.9%)	*p* = 0.527
	Yes, some	10 (9.3%)	4 (7.5%)	6 (11.1%)	
	No	0 (0.0%)	0 (0.0%)	0 (0.0%)	
Loss of activity and/or surgical training	All or almost all	108 (100%)	53 (100%)	54 (100%)	-
Negative effect on residency (scale 0 to 4)	0–2	26 (24.1%)	13 (24.5%)	13 (24.1%)	*p* = 0.956
	3–4	82 (75.9%)	40 (75.5%)	41 (75.9%)	
I wish to prolong the residency training period	Yes	61 (56.5%)	28 (52.8%)	32 (59.3%)	*p* = 0.159
	Undecided.	30 (27.8%)	13 (24.5%)	17 (31.5%)	
	No	17 (15.7%)	12 (22.6%)	5 (9.3%)	
More time dedicated to study (valued at hours/week)	Yes, >5 h more	31 (28.7%)	13 (24.5%)	17 (31.5%)	***p* = 0.022**
	Yes, 3–5 h more	27 (25.0%)	16 (30.2%)	11 (20.4%)	
	Yes, 1–2 h more	34 (31.5%)	21 (39.6%)	13 (24.1%)	
	No	16 (14.8%)	3 (5.7%)	13 (24.1%)	
Decreased in-person working time	Yes	93 (86.1%)	45 (84.9%)	47 (87.0%)	*p* = 0.751
	No	15 (13.9%)	8 (15.1%)	7 (13.0%)	
Duty in another service	Yes	17 (15.7%)	4 (7.5%)	13 (24.1%)	***p* = 0.019**
	No	91 (84.3%)	49 (92.5%)	41 (75.9%)	

## Data Availability

All data and materials would be available if requested.
